# Transmembrane signaling molecules play a key role in the pathogenesis of IgA nephropathy: a weighted gene co-expression network analysis study

**DOI:** 10.1186/s12865-021-00468-y

**Published:** 2021-12-03

**Authors:** Alieh Gholaminejad, Amir Roointan, Yousof Gheisari

**Affiliations:** Regenerative Medicine Research Center, Isfahan University of Medical Sciences, Hezar Jerib Avenue, 81746-73461 Isfahan, Iran

**Keywords:** IgA nephropathy, Weighted gene co-expression network, Differentially expressed genes, Hub genes, Drug target

## Abstract

**Background:**

Immunoglobulin A nephropathy (IgAN) is one of the most common primary glomerulonephritis and a serious health concern worldwide; though still the underlying molecular mechanisms of IgAN are yet to be known and there is no efficient treatment for this disease. The main goal of this study was to explore the IgAN underlying pathogenic pathways, plus identifying the disease correlated modules and genes using the weighted gene co-expression network analysis (WGCNA) algorithm.

**Results:**

GSE104948 dataset (the expression data from glomerular tissue of IgAN patients) was analyzed and the identified differentially expressed genes (DEGs) were introduced to the WGCNA algorithm for building co-expression modules. Genes were classified into six co-expression modules. Genes of the disease’s most correlated module were mainly enriched in the immune system, cell–cell communication and transmembrane cell signaling pathways. The PPI network was constructed by genes in all the modules and after hub-gene identification and validation steps, 11 genes, mostly transmembrane proteins (*CD44*, *TLR1*, *TLR2*, *GNG11*, *CSF1R*, *TYROBP*, *ITGB2*, *PECAM1*), as well as *DNMT1*, *CYBB* and *PSMB9* were identified as potentially key players in the pathogenesis of IgAN. In the constructed regulatory network, *hsa-miR-129-2-3p*, *hsa-miR-34a-5p* and *hsa-miR-27a-3p*, as well as *STAT3* were spotted as top molecules orchestrating the regulation of the hub genes.

**Conclusions:**

The excavated hub genes from the hearts of co-expressed modules and the PPI network were mostly transmembrane signaling molecules. These genes and their upstream regulators could deepen our understanding of IgAN and be considered as potential targets for hindering its progression.

**Supplementary Information:**

The online version contains supplementary material available at 10.1186/s12865-021-00468-y.

## Introduction

IgA nephropathy (IgAN) or Berger’s disease is one of the main causes of kidney failure worldwide [1, 2]. In IgAN, deposition of immunoglobulin A1 (IgA1)-contained complexes in kidneys will cause local inflammations and subsequently hamper the normal function of kidneys, which is filtering of waste out of the blood. Continuation of this condition results in end-stage renal disease (ESRD) in about 40% of patients [3]. Generally, aberrant galactosylation of IgA1 leads to sequential events including the production of autoimmune antibodies (IgG and IgA), binding of these antibodies to the IgA1 molecules, formation of immune complexes and their deposition in the glomerular mesangium [4]. However, despite current knowledge, the underlying molecular mechanisms of IgAN and the details of its pathogenicity are not yet fully understood [5, 6]. Besides, there is a growing number of investigations searching for an efficient treatment for either prevention or treatment of this silent disease. Therefore, further investigations are needed not only to shed a light on the pathogenicity of IgAN disease, but also to discover key elements with therapeutic potentials [7].

Recently, a growing number of systems biology-based studies have investigated high-throughput datasets. Transcriptomics data analysis is one approach, by which researchers could finally catch a big map of expressional alterations in disease versus normal states [6, 8, 9]. Analysis of expression data has become a practical strategy for mining disease-associated genes and novel therapeutic targets in multifaceted disorders like IgAN [7, 10, 11].

Weighted gene co-expression network analysis (WGCNA) is one of the inclusive algorithms for co-expression analysis of high throughput datasets in the R programming language [12]. This algorithm applies a soft threshold to introduce weight values for determining the interaction probabilities among genes. In addition to constructing the weighted co-expression networks and clustering the co-expressed genes in separate modules, another main objective of the WGCNA algorithm is to identify the most correlated modules and genes (intramodular hubs) with an external phenotype [12].

**Fig. 1:**
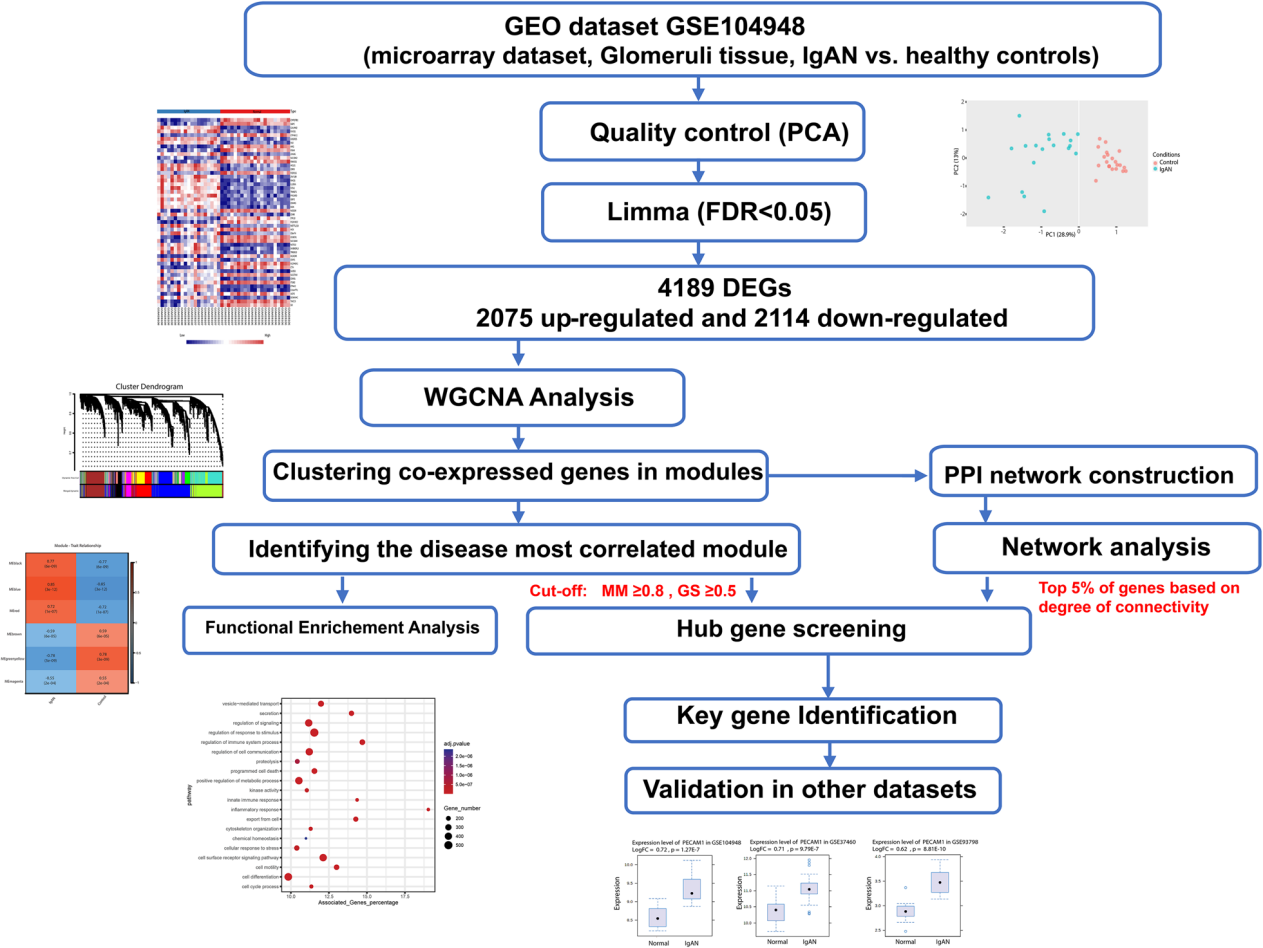
Flowchart of the present study. GEO, Gene Expression Omnibus; PCA, principal component analysis; FDR, false discovery rate; DEGs, differentially expressed genes; WGCNA, weighted gene co-expression network analysis; PPI, protein–protein interaction; MM, module membership

The objectives of this study were to explore the disease-correlated modules, as well as the discovery of the underlying pathogenic pathways and potential therapeutic targets in the IgAN disease. GSE104948 dataset was included the expression data from glomerular tissue of patients with different chronic kidney diseases (Additional file 1: Table S1). In the present experiment, after extracting, quality checking and analyzing the IgAN and living donor samples from this dataset, the intensity values of the identified differentially expressed genes (DEGs) were subjected to WGCNA. Then, the co-expressed modules were subjected to functional analyses and the hub genes were selected based on a constructed protein–protein interaction (PPI) network comprising all DEGs and lists of ranked genes based on module membership (kME) values. Following validation of the hub genes in two other IgAN datasets, the true hub genes (mostly transmembrane proteins) and their top related upstream regulatory elements, including miRNAs and transcription factors (TFs) were identified. Figure 1 shows a flowchart representing the main steps of the work.

## Results

### Preprocessing, analysis, and identification of DEGs: 4189 DEGs were identified based on FDR cutoff

**Fig. 2:**
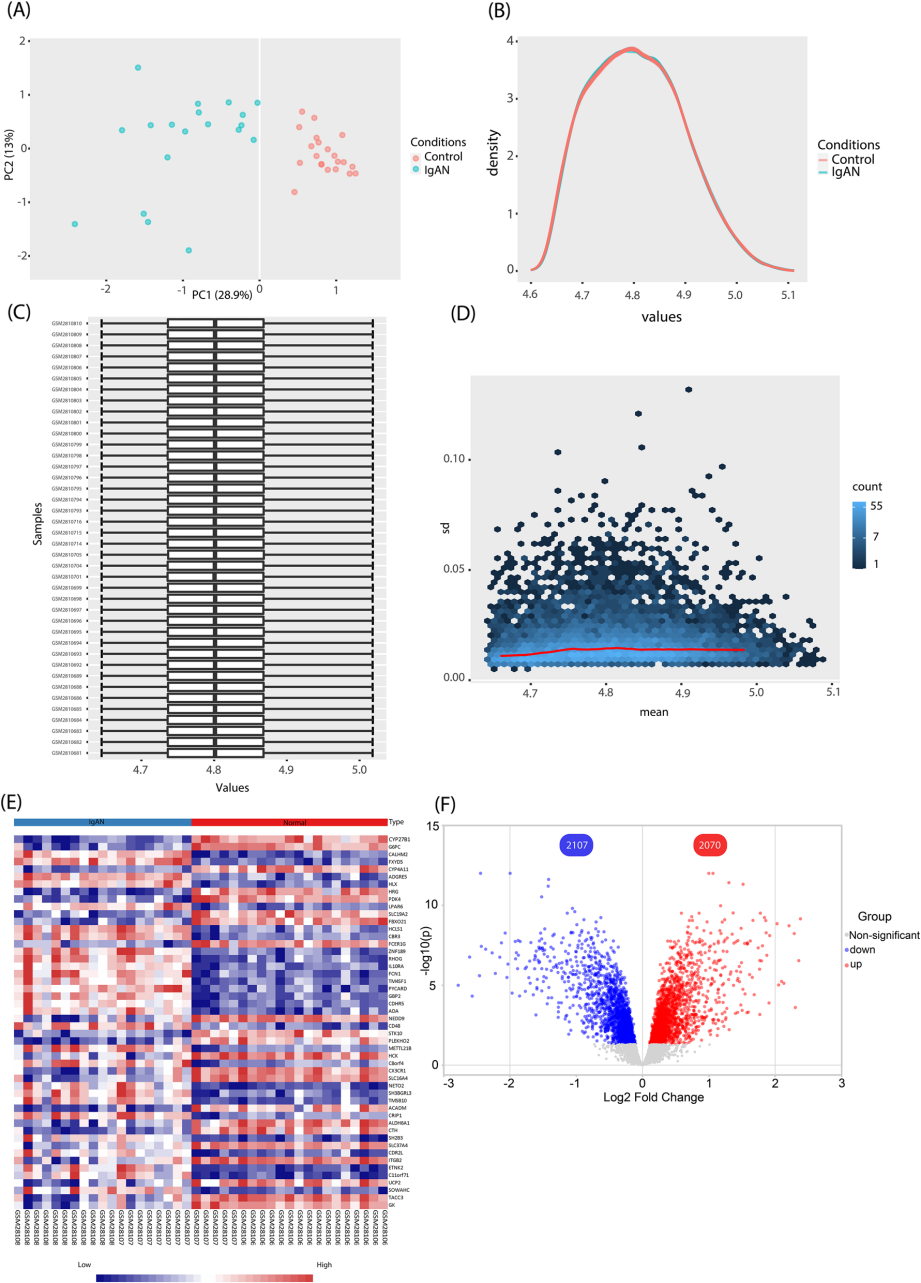
Data preprocessing and processing; Boxes displaying the results of filtering, normalization and processing of the dataset. **A** PCA plot depicting the similarities and differences between the IgAN and healthy samples. **B** The plot of density against log2 of read counts, showing the relative distribution of different counts in each group. **C** Box plot showing the distribution of the normalized samples. **D** Diagnostic plot representing the standard deviation versus mean measures of reads in the samples for each gene. **E** Heatmap representing the top 50 DEGs according to FDR value. **F** Volcano plot of the analyzed dataset

Before the dataset analysis, several preprocessing steps, including PCA and normalization procedures were performed to ensure the accuracy of the main analysis. After performing PCA, 7 outliers were detected among the IgAN samples and removed from the raw dataset file before the analysis (Fig. 2A). Additionally, a normalization procedure (quantile normalization) was conducted to guarantee the similarity of the expression distributions of each sample across the entire dataset (Fig. 2B–D). According to the FDR cutoff, 4189 significant DEGs including 2114 down-regulated and 2075 up-regulated DEGs were detected and subjected to further analysis. A heatmap and a volcano plot representing top 50 DEGs based on FDR value and the results of the analyzed dataset are depicted in Fig. 2E, F.

### Construction of co-expression modules: 6 different co-expressed modules were identified

**Fig. 3:**
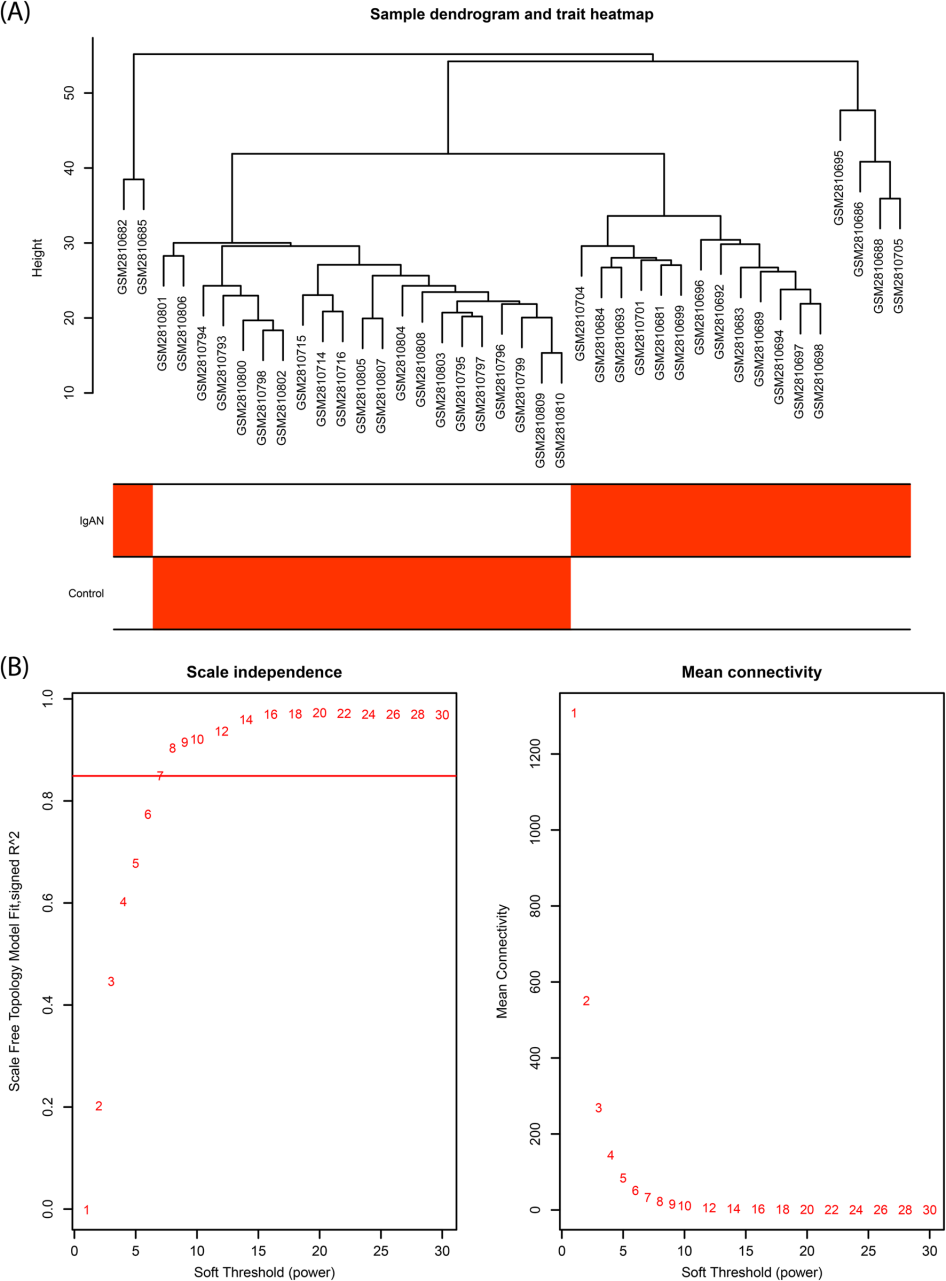
Sample clustering and estimation of the soft-thresholding value. **A** Sample dendrogram and trait heatmap for 18 IgAN and 21 control samples. **B** Plots representing the analysis of scale-free fit index and mean connectivity for different values from 1 to 30. Here, the power of 7 was selected for downstream analysis due to its highest mean connectivity once the scale-free fit index is up to 0.85

**Fig. 4:**
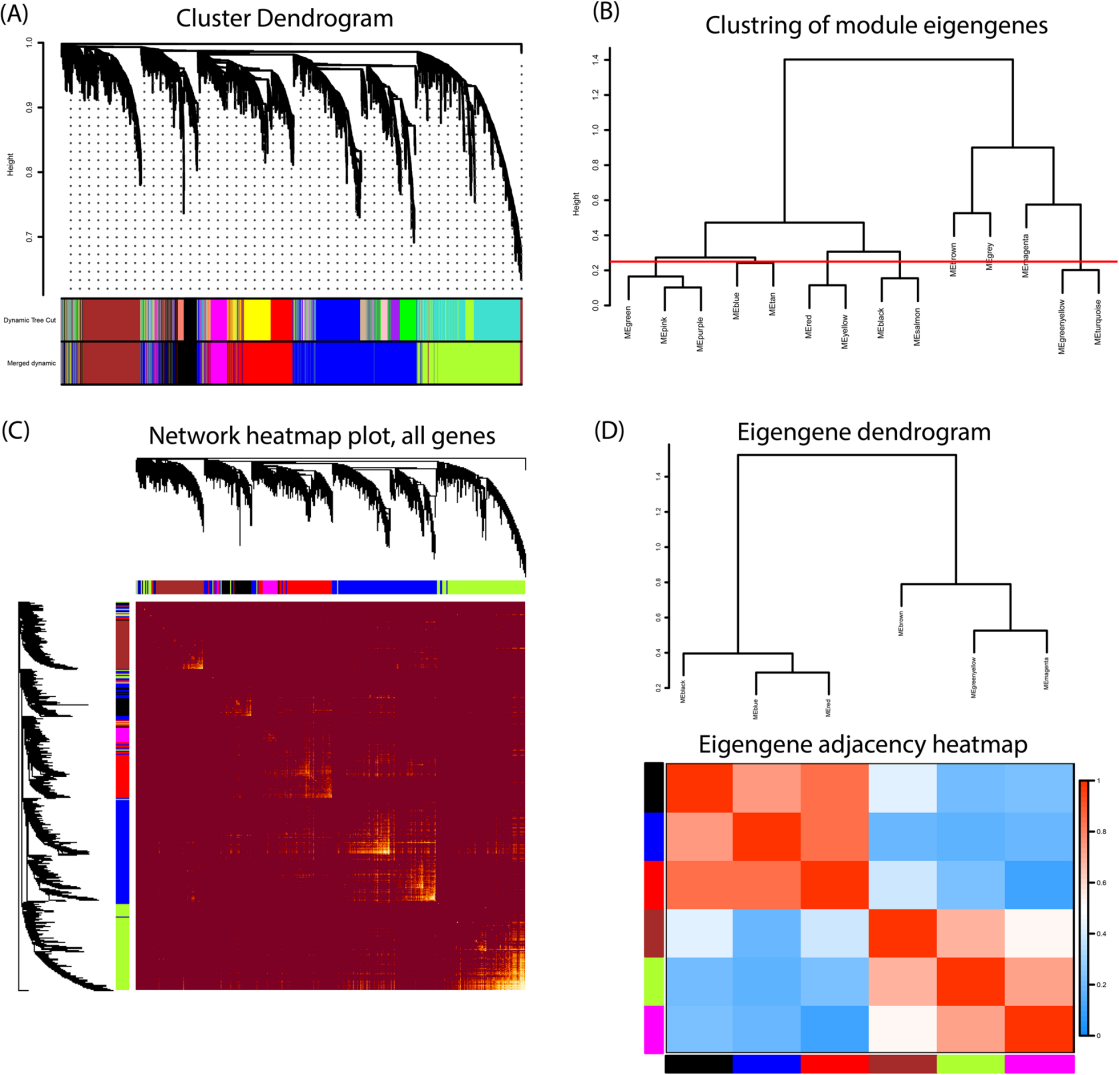
Construction and validation of the co-expression modules. **A** cluster dendrogram depicting the genes (branches) and co-expressed modules (colors); genes are clustered in modules according to 1-TOM. **B** Clustering of module eigengenes and merging the most similar ones (mergeCutHeight = 0.25). **C** Network heatmap plot of all genes showing the module division accuracy. Each row and column belong to a single gene. Red color indicates low adjacencies and progressive yellow color indicates higher adjacencies among genes in the modules. **D** Eigengene dendrogram and eigengene adjacency heatmap representing high (red) and low (blue) adjacencies among the modules

The results of sample clustering, trait heatmap, as well as scale independence, and mean connectivity platforms are shown in Fig. 3A, B. A soft threshold of 7 was selected as the best power to get an approximate scale-free topology. Moreover, after hierarchical clustering and module merging steps, genes were grouped into 6 different co-expression modules including black, blue, brown, green-yellow, magenta, and red (Fig. 4A, B). Figure 4C shows the network heatmap plot representing the accuracy of the module division. The constructed heatmap showed the topological overlap adjacency among genes across the modules, and as we can see there is a higher correlation between the genes in the same module. The eigengene’s clustering dendrogram and eigengene adjacency heatmap also showed the division of the identified modules into two clusters (Fig. 4D).

### Module-phenotype correlation: blue module was identified as the top disease-correlated module

**Fig. 5:**
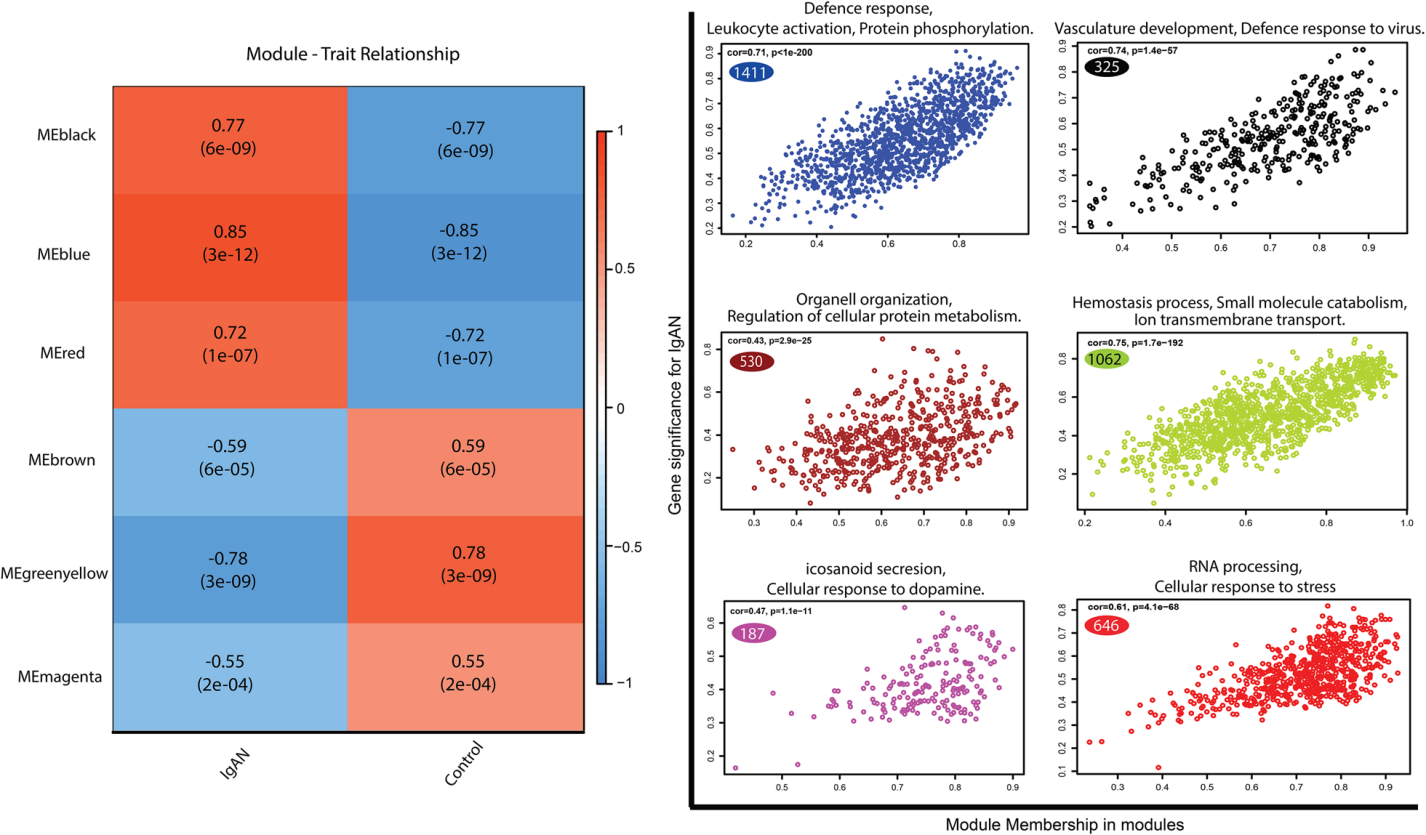
Identification of the clinical related module. **A** The resulted heatmap showing correlations between the modules and the traits (healthy and IgAN individuals). The blue module is selected as the most correlated module with the disease state. **B** Scatter plots representing the module membership (kME or MM) and gene significance (GS) values of the genes in the co-expressed modules. Genes on the upper right part of the plots were selected for hub-gene excavation. The biological process parent terms for each module are shown on the top of each scatter plot

The correlations between each module and two phenotypes (healthy and disease states) were calculated and the most disease-relevant modules were identified. The blue module showed the highest positive correlation with the disease state (r = 0.85; *P* = 3E−12) (Fig. 5A). Black (r = 0.77; *P* = 6E−9) and red (r = 0.72; *P* = 1E−7) were other modules showing a positive correlation with the disease state. Other three modules, including magenta (r = − 0.55; *P* = 2E − 4), brown (r = − 0.59; *P* = 6E−5) and green-yellow (r = − 0.78; *P* = 3E−9) showed negative correlations with the disease state. Scatter plots representing gene significance (GS) vs kME values of all genes in each module are shown in Fig. 5B. A high correlation between the GS and kME values in the blue module (r = 0.71, *p* < 1e−200), suggesting the association of its genes with both the phenotype (disease state) and the module eigengene.

### Functional enrichment analysis: Genes of the blue module were mainly enriched in the immune system and cell-signaling pathways

**Fig. 6:**
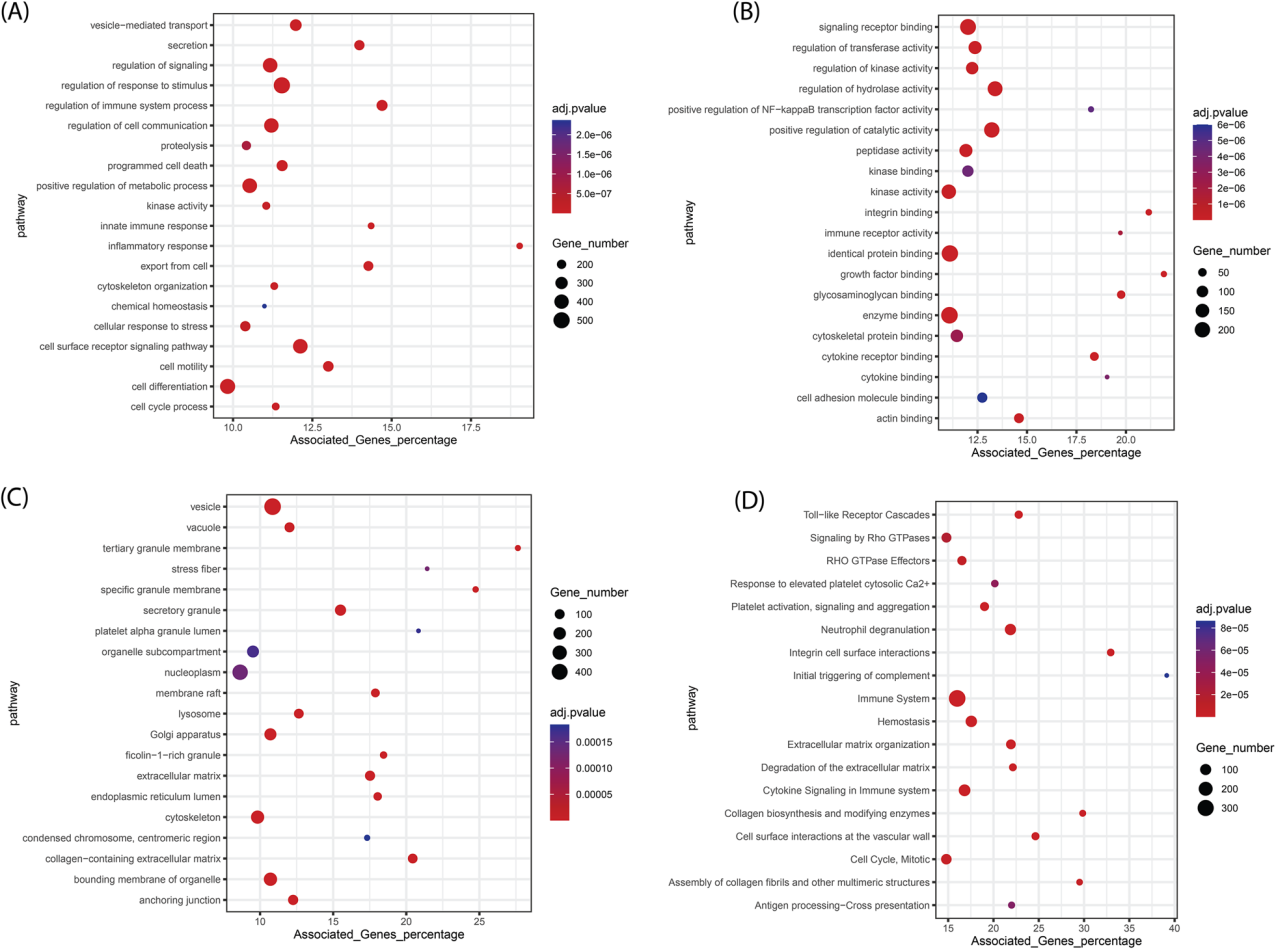
The results of functional analysis for the extracted genes from the blue module. **A** top biological process terms, **B** top molecular function terms, **C** top cellular component terms and **D** top biological pathways

GO and Reactome pathway enrichment analyses were accomplished on the genes of the blue module and the top GO terms and Reactome pathways are shown in Fig. 6. According to the Reactome pathway analysis results, genes in the blue module were mainly enriched in pathways like immune system signaling and extracellular matrix organization. Biological process terms also confirmed the Reactome analysis results, where the primarily enriched terms were related to the regulation of different signaling processes, cell surface receptor signaling pathways, and cell communications. The GO terms of cellular components showed that the blue module genes were mainly enriched in the secretary system, membrane and cytoskeleton. About the molecular function terms, blue module genes were primarily enriched in signaling receptor binding, kinase activity, enzyme binding, and cytoskeletal protein binding. The parent biological process GO terms of other modules are also shown in Fig. 5B and listed in Table 1.

**Table 1:** The biological process parent terms for all co-expressed modules

Module	Term	count	% Associated Genes	*p*-value
Black	Vasculature development	47	6.18	1.24E−14
	Defense response to virus	23	8.48	2.59E−10
	Leukocyte proliferation	18	5.35	1.89E−05
	Regulation of cell adhesion	38	4.99	2.59E−09
Blue	Leukocyte activation	241	16.88	2.58E−32
	Defense response	273	14.62	3.60E−26
	Inflammatory response	150	19.03	5.24E−24
	Regulation of multicellular organismal process	350	12.44	3.56E−21
	Protein phosphorylation	210	12.10	1.15E−09
Brown	Organelle organization	180	4.25	1.33E−10
	Regulation of cellular protein metabolic process	124	4.76	5.19E−10
	Circulatory system development	64	5.62	5.81E−08
	Intracellular transport	85	4.62	1.83E−06
Green yellow	Small molecule catabolic process	135	28.06	1.96E−55
	Ion transmembrane transport	176	11.50	3.65E−21
	Homeostatic process	191	9.26	6.24E−13
	Xenobiotic metabolic process	32	23.70	2.00E−12
	Circulatory system process	78	11.94	1.74E−10
Magenta	Cellular response to dopamine	6	6.31	2.27E−04
	Icosanoid secretion	4	8.33	9.30E−04
Red	RNA processing	99	9.96	6.20E−22
	RNA localization	31	11.96	1.59E−09
	Cellular response to stress	115	5.28	2.21E−06

### PPI network construction and hub-gene excavation: 16 hub genes were identified among all genes in the co-expressed modules

**Fig. 7:**
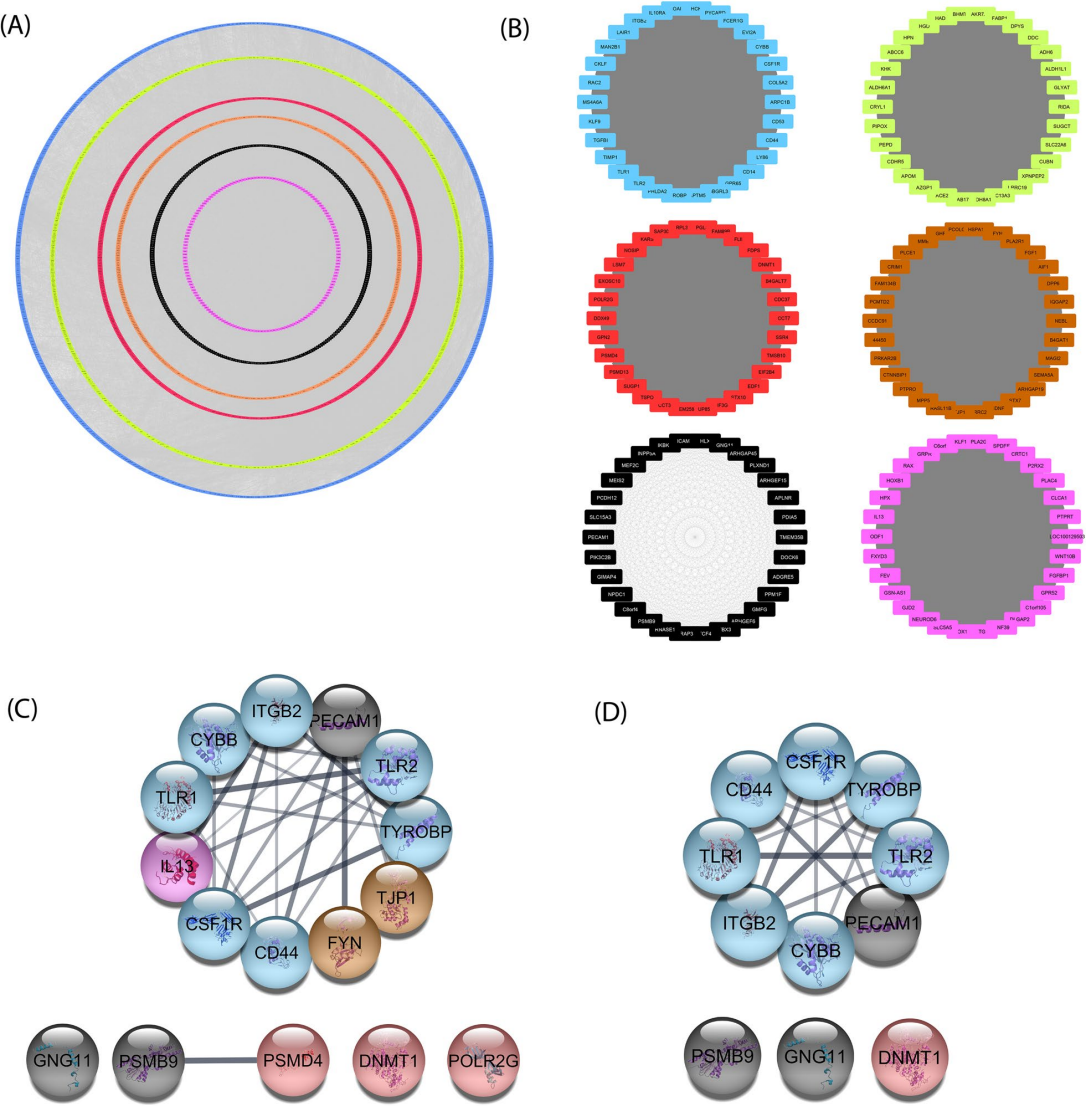
PPI network construction and hub gene selection. **A** The constructed PPI network for all the genes in modules. **B** Top 30 genes of each module based on module membership values (kME > 0.8). **C** 16 identified hub genes. Colors representing the module colors (**D**). 11 validated hub genes (true hub genes) using the highly correlated genes based on gene significance (GS) and module membership (MM) values. Hub genes were the ones among both the top 5% of genes in each module in the PPI network (based on degree centrality) and top 30 genes ranked by kME value. The constructed PPI network is accessible at network data exchange (NDEx) server by clicking on the URL: “https://public.ndexbio.org/#/network/5c60dee6-66dd-11eb-9e72-0ac135e8bacf?accesskey=7af2e78864f10bfa6107789793d7581214d6c1485e4cd566975fbe8df4469a30”

The PPI network with 4031 nodes and 81,259 edges was constructed using all the genes in 6 modules (Fig. 7A). Moreover, after ranking the genes based on kME value, the top 30 genes in each module were extracted from the WGCNA algorithm and visualized in Cytoscape (Fig. 7B). Considering both the kME value and the degree of connectivity in the PPI network, 16 genes were selected as hub genes (Fig. 7C). The identified hub genes included colony-stimulating factor 1 receptor (*CSF1R*), cytochrome b-245 beta chain (*CYBB*), TYRO protein tyrosine kinase binding protein (*TYROBP*), integrin subunit beta 2 (*ITGB2*), toll-like receptor 1 (*TLR1*), toll-like receptor 2 (*TLR2*), and *CD44* from the blue module, G protein subunit gamma 11 (*GNG11*), proteasome 20S subunit beta 9 (*PSMB9*), and platelet and endothelial cell adhesion molecule 1 (*PECAM1*) from the black module, DNA methyltransferase 1 (*DNMT1*), RNA polymerase II subunit G (*POLR2G*) and proteasome 26S subunit, non-ATPase 4 ( *PSMD4*) from the red module, interleukin 13 (*IL13*) from the magenta module and tight junction protein 1 (*TJP1*) and FYN proto-oncogene (*FYN*) from the brown module (Table 2). According to our strategy, most of the hub genes were detected in the blue module and the green-yellow module had no hub gene. All of the spotted genes had kME value > 0.9 and a relative high GS value for IgAN disease, which confirmed their system supremacy and potential roles in IgAN pathogenesis.

**Table 2:** All the identified and validated hub genes in the datasets. True hub genes were defined as verified ones in two other IgAN datasets

No	All hub genes	Modules	MD^a^ (GSE104948)	VD1^b^ (GSE93798)	VD2^c^ (GSE37460)	True hub genes
1	Colony stimulating factor 1 receptor (CSF1R)	Blue	✓	✓	✓	✓
2	Cytochrome b-245 beta chain (CYBB)	Blue	✓	✓	✓	✓
3	TYRO protein tyrosine kinase binding protein (TYROBP)	Blue	✓	✓	✓	✓
4	integrin subunit beta 2 (ITGB2)	Blue	✓	✓	✓	✓
5	Toll-like receptor 1 (TLR1)	Blue	✓	✓	✓	✓
6	Toll-like receptor 2 (TLR2)	Blue	✓	✓	✓	✓
7	CD44	Blue	✓	✓	✓	✓
8	Proteasome 20S subunit beta 9 (PSMB9)	Black	✓	✓	✓	✓
9	G protein subunit gamma 11 (GNG11)	Black	✓	✓	✓	✓
10	Platelet and endothelial cell adhesion molecule 1 (PECAM1)	Black	✓	✓	✓	✓
11	DNA methyltransferase 1 (DNMT1)	Red	✓	✓	✓	✓
12	RNA polymerase II subunit G (POLR2G)	Red	✓	-	-	-
13	Proteasome 26S subunit, non-ATPase 4 (PSMD4)	Red	✓	✓	-	-
14	Tight junction protein 1 (TJP1)	Brown	✓	-	✓	-
15	FYN proto-oncogene (FYN)	Brown	✓	-	✓	-
16	Interleukin 13 (IL13)	Magenta	✓	-	✓	-

^a^Main dataset

^b^Validation dataset 1

^c^Validation dataset 2

### Hub-gene validation: 11 hub genes were validated and introduced as true hub genes

**Fig. 8:**
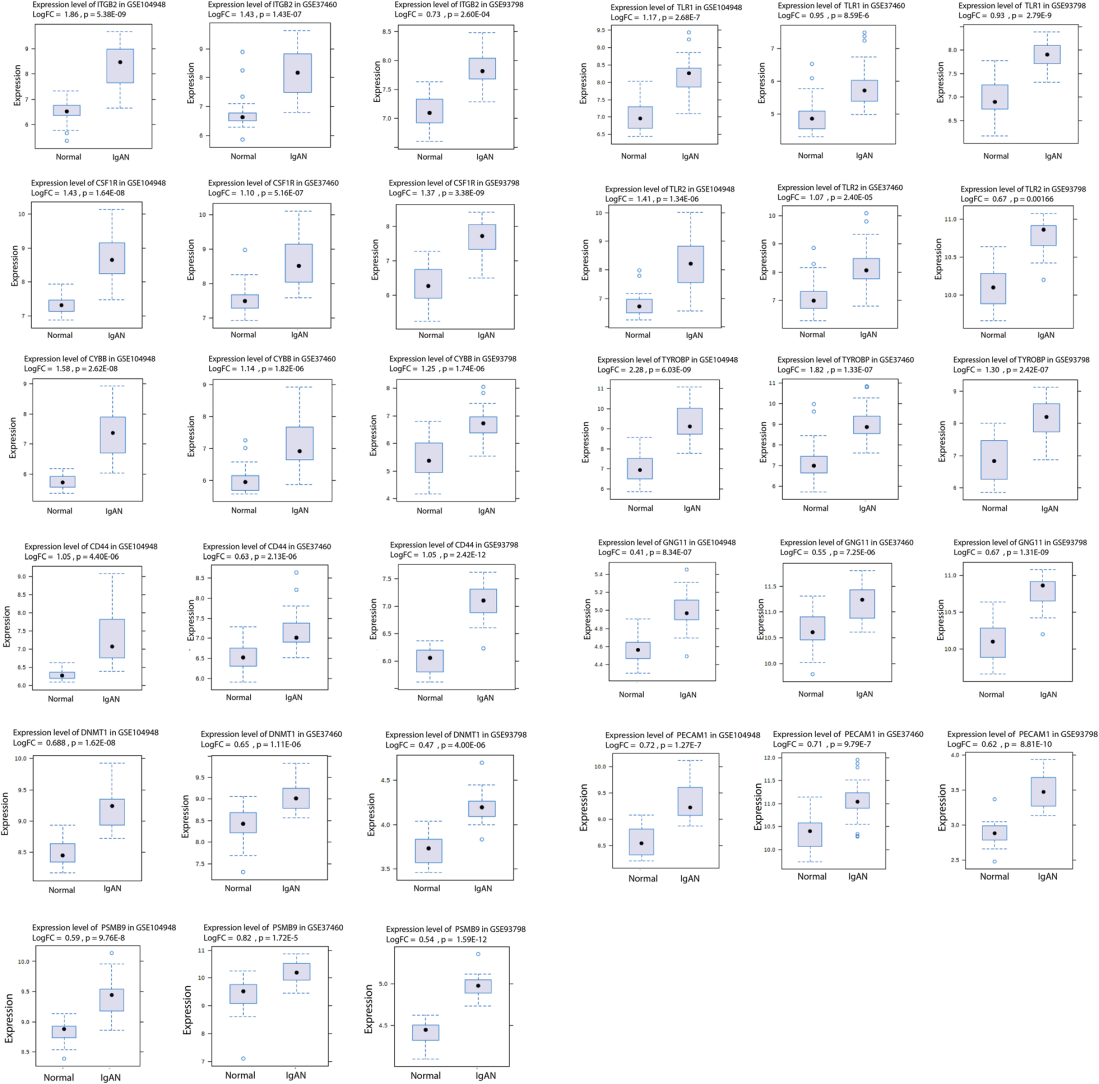
Differentially expressed levels of the introduced hub genes (true hub genes) between IgAN and normal in different GEO datasets. In three datasets, all the hub genes showed an upregulated pattern in the disease state

To verify the differentially expressed profiles of the 16 identified hub genes in other IgAN related datasets, GSE93798 and GSE37460 were analyzed. In this step, 11 out of 16 hub genes were identified as DEGs in two other datasets with upregulation patterns and close log2FC values (Fig. 8, Fig. 7D). The 11 validated hub genes were introduced as true hub genes (Table 2).

### MiRNA-TF enrichment study: has-miR-129–2-3p, hsa-miR-34a-5p, hsa-miR-27a-3p and STAT3 were the top up-stream regulators of the hub genes

**Fig. 9:**
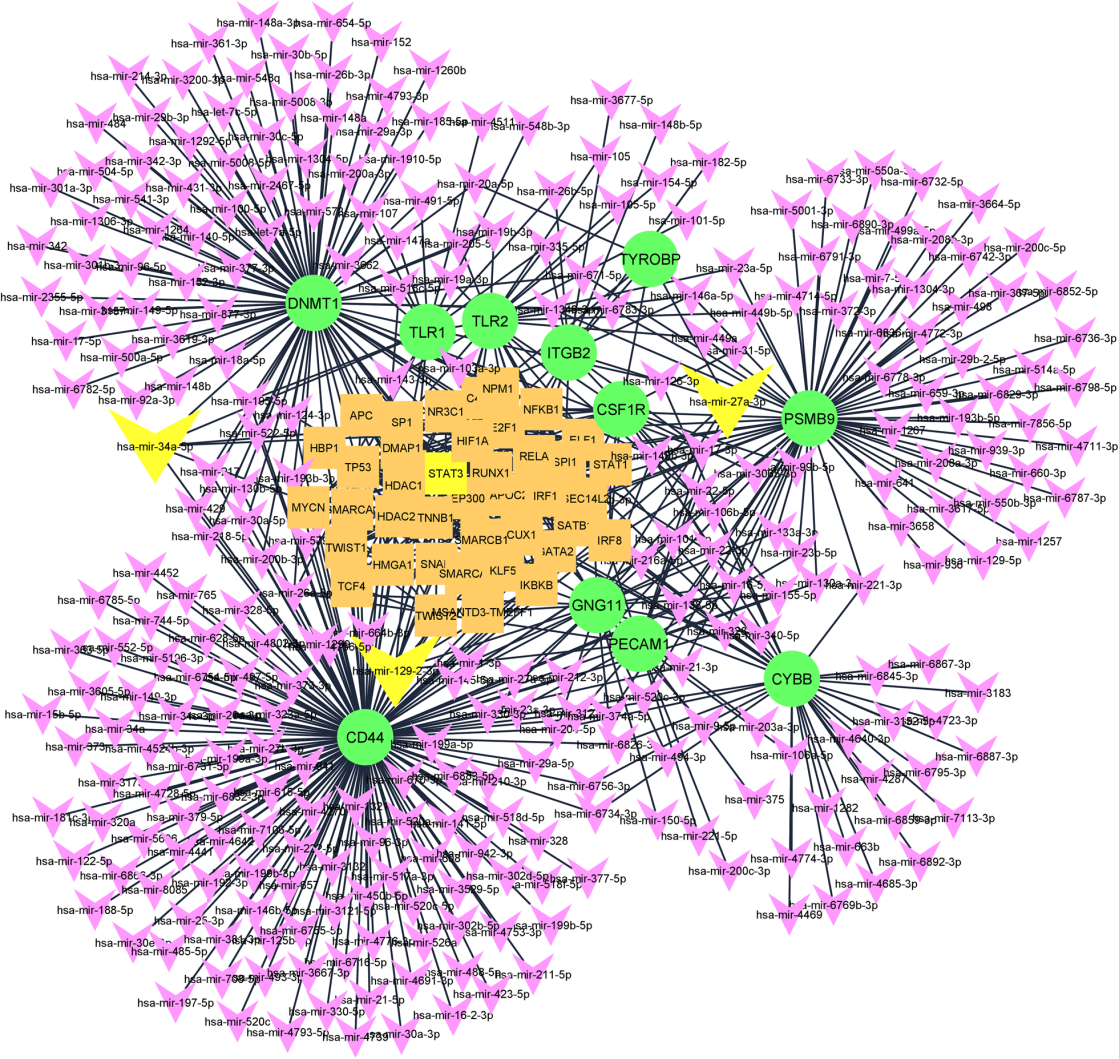
The constructed regulatory network comprising of 11 true hub genes and the enriched miRNAs, and transcription factors. CD44 was identified as the most affected hub-gene, as well as *hsa-miR-129-2-3p*, *hsa-miR-34a-5p*, *hsa-miR-27a-3p* and STAT3 were recognized as top miRNA and TF molecules, targeting the true hub genes. (Network link at NDEx: https://public.ndexbio.org/#/network/5999d4b1-6853-11eb-9e72-0ac135e8bacf?accesskey=2beea546b0619da5261dafb9f9e3233b61216b074a44751d46966a74b80d607c)

To recognize upstream regulatory elements affecting the expression of the true hub genes, a regulatory network containing true hub gene’s interrelationships, the enriched miRNAs and TFs was constructed and analyzed (Fig. 9). The network consists of 350 nodes including 11 hub genes, 298 miRNA, and 41 TFs. In this network, *CD44* was the most affected hub-gene by both miRNAs and TFs. Also, *hsa-miR-129-2-3p*, *hsa-miR-34a-5p*, *hsa-miR-27a-3p*, and *STAT3* were the top 3 miRNAs and TF targeting the true hub genes.

## Discussion

So far, several datasets containing the human glomeruli mRNA expression profiles from IgAN individuals were produced and subjected to analysis by different strategies. The main objective of such investigations is to clarify the disease’s basic molecular mechanisms, along with introducing key drivers in the pathogenesis of the disease. For instance, in one experiment after reanalysis of two IgAN datasets, including GSE73953 and GSE93798, tumor nephrotic factor (*TNF*) and mitogen-activated protein kinase (*MAPK*) pathways were introduced as the key involved pathways in the IgAN pathogenesis [13]. Similarly, in another experiment, after analysis of three datasets, including GSE37460, GSE93798 and GSE104948, Miraji et al., revealed the association of “extracellular matrix receptors interaction pathways”, “extracellular matrix expansion” and “inflammatory mechanisms” with the pathogenesis of IgAN [14]. By construction of a PPI network among the overlapped DEGs and considering the degree of connectivity between the genes, the authors introduced several hub genes with therapeutic potentials including *FN1*, *ITGB2*, *FCER1G* and *PTPRC* [14].

Despite these findings, it seems that more excavations, especially by means of potent and comprehensive strategies are needed to cut deeper and reach more exhaustive and robust results, especially in the case of complex diseases like IgAN. In the present study, we applied WGCNA as an advanced and comprehensive algorithm to construct gene co-expression networks, exploring modules, and identifying disease correlated modules and genes in IgAN related samples. At first, the IgAN dataset GSE104948 was subjected to several pre-analysis steps including normalization and outlier removal and after analysis using the ‘limma’ R package, the identified DEGs were subjected to co-expression network construction. After clustering the co-expressed genes and module detection procedure, biological process enrichment analyses and hub-gene identification steps were performed for all the co-expressed modules. Of all the genes in modules that showed a high kME value, as well as a high degree of centrality in the PPI network, 16 genes were identified and after the validation procedure, 11 genes were introduced as true hub genes. Notably, most of the true hub genes (7 out of 11) were coming from the blue module, which was also spotted as the top disease correlated module. As far as we know, there is no WGCNA analysis study on this dataset. In addition, for hub-gene identification, the present experiment applied a more comprehensive strategy, when compared to other similar experiments. Researchers usually consider the degree of connectivity in either a PPI network of genes with high kME values in a module or a PPI network including genes of only one module [15, 16]. However, to keep the holistic view of systems biology, we mined the hub genes, considering both the list of top genes in the co-expressed modules, as well as a PPI network comprising of the genes in all modules. Moreover, a regulatory network that included the hub gene’s related miRNAs and TFs was constructed to catch a comprehensive view of the disease pathogenesis.

GO and Reactome pathway data are two categories of biological materials essential for understanding mechanisms underlying the disease processes. The immune system signaling, inflammatory responses, cellular communication and extracellular matrix-related pathways were among the top enriched pathways for genes in the blue module. Such results were in line with previous experiments showing the association of innate immune responses and inflammatory reactions with IgAN [17, 18]. Neutrophil degranulation, cytokine signaling and inflammatory responses were other enriched terms that confirmed the strong link of immune-related pathways with the pathogenesis of IgAN.

Toll-like receptors (*TLRs*) are well-known components of the innate immune system. Similar to previous experiments [19, 20], the results of the present study also revealed the upregulation of these receptors in the IgAN samples. After binding to their ligands (pathogen-associated molecular patterns), *TLRs* trigger various immune signal cascades in order to promote immune system activation. Nevertheless, these receptors might cause glomerular damage through the induction of inflammatory cytokines in IgAN patients [21]. In the present study, *TLR1* and *TLR2* were introduced as two hub genes with potential involvement in IgAN pathogenesis. Co-expression and interaction of these two receptors finally lead to the activation of NFκB, as well as different immune cells like B cells, dendritic cells, mast cells, NK cells and keratinocytes [22]. Accordingly, these receptors might be considered as potential therapeutic targets aimed for the attenuation of immune responses in IgAN. Although the potential role of TLRs has not been extensively investigated in the IgAN pathogenesis, they have been shown to play an important role in the induction of inflammatory responses in other kidney diseases [22].

*CYBB*, *CSF1R*, *TYROBP*, *ITGB2*, and *CD44* were other identified hub genes in the blue module that function either as regulatory elements or signal transducers in the regulation of immune responses. Despite the *CYBB*, all of the identified hub genes in the blue module were transmembrane proteins participating in cell–cell communication and/or signal transduction.

The *CYBB* gene is responsible for coding cytochrome b-245, the key subunit of the *NADPH* oxidase, which is the membrane-bound oxidase of phagocytes. Host defense through regulation of antigen processing and presentation, as well as regulation of phagocytes and neutrophils is the main function of *NADPH* oxidase [23].

*CSF1R* is a transmembrane protein acting as a cell-surface receptor for *CSF1* and interleukin-34. The crucial role of *CSF1R* in the regulation of survival, proliferation and differentiation of mononuclear phagocytes like macrophages and monocytes has been shown by previous studies [23]. Upon ligand binding, *CSF1R* enhances the release of proinflammatory chemokines and therefore has a significant role in inflammatory processes [24, 25]. Similarly, *TYROBP* or *DAP12* encodes a transmembrane signaling polypeptide, which acts as a signal transduction element. Activation of additional tyrosine kinases, cell activation, integrin-mediated neutrophil activation and formation of inflammatory cytokines are some revealed functions of this transmembrane protein [26, 27].

Cell surface interactions and extracellular matrix organizations were other enriched terms for the blue module genes. These terms are not irrelevant to the immune system-related pathways, since cell–cell and cell–matrix intercommunications are vital in triggering inflammatory responses and activating immune cells. Here, functions of *ITGB2* and *CD44* as two identified hub genes, as well as Rho GTPases could be of attention, due to their roles in cytoskeletal organization, cellular communication, and immune signaling.

Rho GTPases are well-known for their regulatory roles in cytoskeleton dynamics, cell movement, cellular signaling, phagocytosis and inflammation [28, 29]. Based on some reports, Rac-1 and RhoA, as two key members of Rho GTPases, are listed as two mediators of podocyte dysfunction and therefore, their inhibition might be beneficial for handling chronic kidney diseases (CKDs) [29, 30]. Despite such findings, there is a limited number of studies concerning the involvement of Rho GTPases in IgAN pathogenesis. Considering their roles in signaling transduction and cytoskeleton organization, these molecules could be candidates of more investigations exploring their involvement in IgAN pathogenesis.

As part of integrin heterodimers, *ITGB2* is participating in both cell adhesions and surface-mediated signaling. According to previous experiments, there is a negative correlation between *ITGB2* and eGFR in patients with CKD [31]. In addition to cell surface interactions, *ITGB2* has also been shown to be involved in the regulation of immune system-related pathways like toll-like receptors cascades, neutrophil degranulation, and interleukin signaling pathways [32, 33]. However, so far, a limited number of studies have pointed to the possible role of *ITGB2* in IgAN pathogenesis and it seems that more investigations are needed to shed a light on this issue [34].

*CD44* was another marked hub-gene showing an up-regulated pattern in IgAN patients. *CD44* which is a well-known cell-surface glycoprotein is involving in diverse biological pathways like hematopoiesis, cell adhesion, proliferation, migration, and lymphocyte activation [35]. Due to various physiological activities of *CD44*, so far, involvement of this glycoprotein has been shown in a wide range of disorders, including vascular disease, arthritis, infections, and cancers [36]. Considering the contribution of *CD44* in cell–cell and cell–matrix connections, same as *ITGB2*, this protein might play a role in immune system signaling and triggering the inflammatory cascades, thus could be considered as a target of more investigations aimed for attenuation of immune responses in IgAN. According to previous experiments, up-regulation of *CD44* in glomerular visceral epithelial cells could be a sign of active injury in glomerular and kidney dysfunction in IgAN patients [37]. In another experiment, a significant correlation was observed between the expression of *CD44* in glomerular and tubulointerstitial and renal damage degree in IgAN individuals [38]. Therefore, in addition to its therapeutic potential, *CD44* may be regarded as a reliable marker of IgAN pathogenesis.

Other identified hub genes in the present experiment included proteasome 20S subunit beta 9 (*PSMB9*), G protein subunit gamma 11 (*GNG11*), platelet and endothelial cell adhesion molecule 1 (*PECAM1*) and DNA methyltransferase 1 (*DNMT1*) were coming from the black and red modules. Genes of the black module were mainly enriched in vasculature development, defense response to virus, leukocyte proliferation, and regulation of cell adhesion.

Considering the functions of *GNG11*, as a member of the heterotrimeric G protein complex and *PECAM1*, as a receptor on platelets, monocytes, granulocytes, macrophages, lymphocytes and endothelial cells, here again, we can see the contribution of these two hub genes in intercommunication and stimulation of immune cells [39, 40]. Likewise, *PSMB9* as an essential subunit of the 20S proteasome complex, is playing a key role in antigen processing, generation of class I binding peptides and finally activation of CD8 T cells and NF-κB pathway [41, 42]. As far as we know, there is no investigation pointing to the potential role of the above-mentioned proteins in the pathogenesis of IgAN. However, their contribution in immune cell intercommunication and activation could imply their association with autoimmune diseases like IgAN.

Another identified hub-gene was *DNMT1*, which is a well-known epigenetic factor transferring methyl groups to CpG structures in DNA. Based on some findings, genomic factors also could have an impact on the pathogenicity of IgAN [43, 44], and in this context, methylation of DNA by DNMT1 could be of attention. Although, we found no investigation pointing to the role of *DNMT1* in the IgAN pathogenesis, inhibition of this epigenetic factor in the kidneys of diabetic nephropathy db/db mice model led to podocyte protective effects [45]. Hindering the progression of kidney diseases like IgAN by inhibition of DNA methylation could be an innovative therapeutic idea and at this point, DNMT1 could be a potential target.

Taking some more steps towards translational medicine, we also performed miRNA and TF enrichment study and constructed a network comprising of true hub genes, and their related miRNAs and TFs. Up to now, the position and diverse roles of miRNAs have been verified in various diseases, where their mutations or aberrant expressions could trigger or augment a condition. Such connections between the disease phenotype and miRNA dysfunction/dysregulation have raised the idea that miRNA modulation might change the disease progression. In the present study, *hsa-miR-129-2-3p*, *hsa-miR-34a-5p*, and *hsa-miR-27a-3p* were identified as top up-stream regulators of the hub genes. The involvement of *hsa-miR-27a-3p* and *hsa-miR-34a-5p* in regulating inflammatory responses in some kidney diseases like diabetic nephropathy and IgAN have been shown previously [46, 47]. However, the present experiment is the first one indicating the potential regulatory role of has-*miR-129* in the pathogenesis of IgAN. This miRNA has been shown to orchestrate different genes involving in cell proliferation, cell cycle, apoptosis, DNA methylation, and metastasis. Moreover, its aberrant expression was observed in different cancers, pointing to the potential role of this miRNA in cancer development [48].

In the case of TFs, signal transducer and activator of transcription 3 (*STAT3*) was identified as top TF, affecting the expression of the hub genes. *STAT3* is expressed in different cell types, like leukocytes and fibroblasts and following activation by interleukin-6, this TF could target genes that induce the production of growth factors, cytokines, and ECM components [49]. Since these elements are contributing to tissue fibrosis, inhibition of *STAT3* activation (phosphorylation) might hinder the process of kidney fibrosis in CKDs [50]. It seems that *STAT3* and the three enriched miRNAs are other pieces of the big network orchestrating the inflammatory responses in the IgAN disease. Though, further investigations are required to validate this hypothesis.

All in all, it should be mentioned that the main limitation of this study was the absence of an experimental section, examining the expression of the identified hub genes in IgAN samples. However, to compensate for this limitation we performed a validation procedure in other IgAN datasets. Another limitation of this work was the absence of glomerulonephritis samples as another control group that could confirm or reject the specificity of the obtained results for the IgAN disease.

## Conclusions

In conclusion, the introduced genes, miRNAs and pathways could deepen our understanding of the underlying molecular mechanisms of IgAN disease and could be the targets of more investigations as potential therapeutic targets. Most of the identified hub genes were transmembrane proteins functioning in cell–cell intercommunications and cell signaling procedures in immune system-related pathways.

## Methods

### Dataset qualification and analysis

The IgAN dataset GSE104948 related to the glomeruli tissue of IgAN patients with 21 control and 27 case samples was downloaded from Gene Expression Omnibus (https://www.ncbi.nlm.nih.gov/geo/). Before analysis, principal component analysis (PCA) was performed to detect and remove possible outliers. Networkanalyst online tool (http://www.networkanalyst.ca) was utilized for sample normalization and dataset analysis. Furthermore, probes related to multiple genes were removed and the mean values of probes were considered as gene’s expression values for those genes matching with multiple probes. Linear Model for Microarray Analysis (Limma) was selected for the analysis procedure and significant DEGs were identified based on false discovery rate (FDR) cutoff < 0.049.

### WGCNA: Construction of gene co-expression networks

WGCNA algorithm in R programming environment was utilized for detection of co-expressed modules. Raw data related to the identified DEGs was introduced to the WGCNA algorithm. After sample clustering, ‘pickSoftThreshold' function in the WGCNA package was applied to screen the best soft-threshold power from 1 to 30. An appropriate soft threshold was selected according to the degree of independence and mean connectivity values. Several steps including the construction of adjacency matrix, calculation of topological overlap matrix (TOM), module construction and dynamic branch cutting with a merging threshold of 0.25, were performed to obtain the co-expression modules. Following parameters were designated for identification of the co-expression modules: “soft-threshold power = 7, minModuleSize = 30, mergeCutHeight = 0.25. A heatmap based on TOM dissimilarity was drawn to describe the adjacencies among genes in the identified modules and to verify the module’s division consistency. After the assignment of samples as normal or IgAN, the correlation between the co-expression modules and these two states (traits) was calculated and the most correlated module was identified.

### Functional enrichment analyses

The extracted genes from the module of interest were subjected to functional enrichment analyses using the functional analysis option in the ClueGO module (version 2.5.7) [51] in Cytoscape software (version 3.8.2) [52]. In the ClueGO module, Reactome pathway and gene ontology (GO) annotation data were used for pathway enrichment and GO enrichment analyses. The significant enrichment threshold for all the analyses was set as *P* < 0.05 and Bonferroni step down was selected for pv correction. Other options in the ClueGO module remained as default. The parent GO terms of biological process also were obtained for the genes in all the modules. In this part, at first, and ClueGO module was utilized for GO and Reactome pathway enrichments, and then, Revigo webserver (http://revigo.irb.hr/), was applied for selection of the parent terms.

### Selection of candidate hub genes

The selection of candidate hub genes was performed using both Module membership (kME) and degree connectivity of the genes in a constructed PPI network. The kME is simply defined as the correlation of a gene with the module eigengene [12]. In this part, following the construction of a PPI network using all genes in the co-expressed modules, the top 5% of genes with a high degree of connectivity in each module were detected and listed. Likewise, considering the kME value (kME > 0.8), top 30 genes of each module were extracted and finally, hub genes were presented as the genes with the highest degree of connectivity and kME values. STRING database (confidence > 0.4) was applied for the construction of the PPI network [53]. Moreover, network visualizations were performed using Cytoscape (v.3.8.2; cytoscape.org/).

### Hub-gene validation in other IgAN related datasets

The expression pattern of the identified hub genes was checked in GSE37460 and GSE93798 as two other IgAN array sets. Both of the selected datasets contained microarray data from glomeruli tissue of IgAN and healthy individuals. GSE37460 included data from 27 healthy and 27 IgAN individuals and GSE93798 contained data from 22 healthy and 20 IgAN patients. These two datasets were analyzed by the same protocol as mentioned before for the main dataset GSE104948. The details of all the analyzed datasets and the results of their analysis are provided in Additional file 1: Table S1.

### MiRNA-Transcription factor enrichment study

In order to find the upstream regulators of the identified hub genes, and taking some more steps toward translational medicine, a regulatory network comprising of the identified hub genes and their associated miRNAs and TFs was constructed. In this part, miRTarBase (Release 7) [54] and TRRUST (Version 2) database [55] were used for identifying the most related miRNAs and TFs. Network construction was conducted using Cytoscape software.

## Supplementary Information

**Additional file 1:** The details of all the analyzed datasets and the results of their analysis.

## Data Availability

The analyzed dataset by the present study is available in the GEO repository, [https://www.ncbi.nlm.nih.gov/geo/query/acc.cgi?acc=GSE104948].
